# Supplementary material for: Estrogen and progesterone-mediated differential modulation of CD4+ T-cell pathogenicity in rheumatoid arthritis
Source: Front Immunol. 2026 May 5;17:1813310. doi: 10.3389/fimmu.2026.1813310 (PMC13183634; doi:10.3389/fimmu.2026.1813310)
Supplement: Supplementary file 1 [file SupplementaryFile1.docx]

## ERα PR


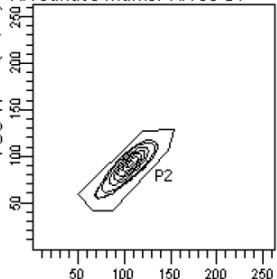

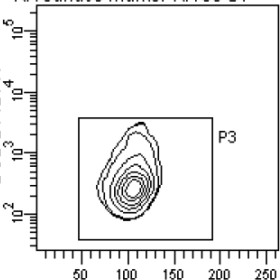

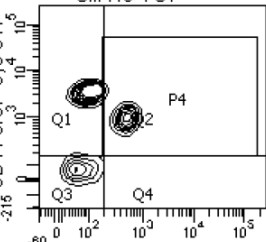

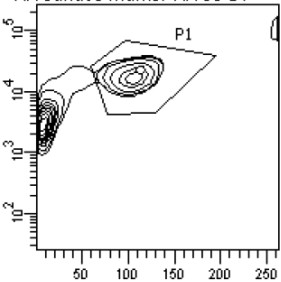

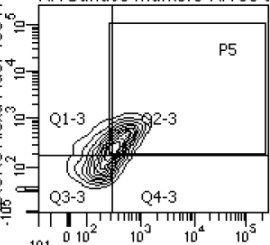


FSC-A

FSC-A

FSC-A

CD4

**CCR6**


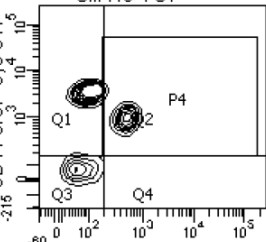


SSC-A

FSC-H

DCD

CD3

**CXCR3**

### CD4


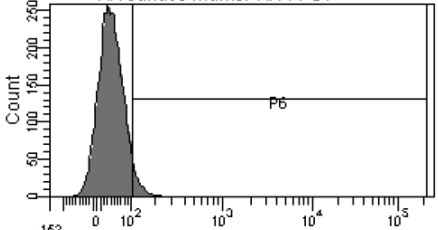

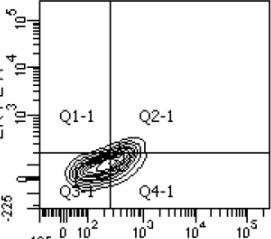


CD3

**ER**α

**ER**α **CXCR3**

**PR CXCR3**

**CCR6**

**CCR6**


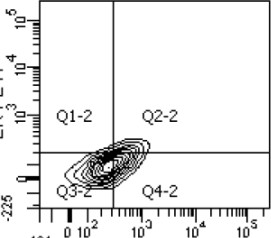

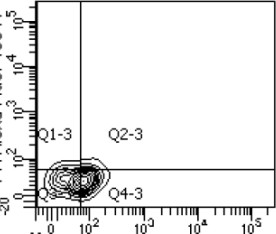


**PR**


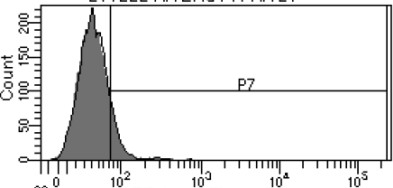
**Supplementary figure 1. Gating strategy for sex hormone receptors, ER**α **and PR expression in RA and HC CD4+ T cells.** Representative flow cytometry plots shows lymphocyte population is gated first, followed by singlet population. Live cells are gated using Zombie violet dye. The dual positive CD3+ CD4+ T cells are selected to analyze for ERα and PR expression. Next, T-helper subset analysis was done based on surface marker CXCR3 for Th1 and CCR6 for Th17. ERα and PR was then analyzed on these T- helper subsets. Also, dual positive CXCR3+CCR6+ compartment was analyzed for ERα and PR expression.


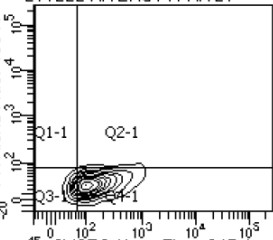


**PR**

**ER**α


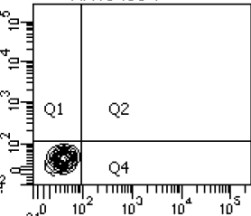


V

#### IFNγ


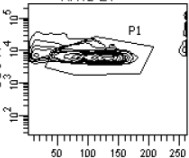


FSC-A


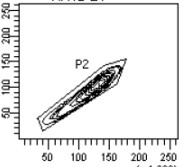


FSC-A


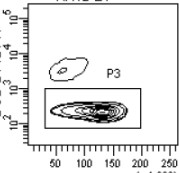


FSC-A


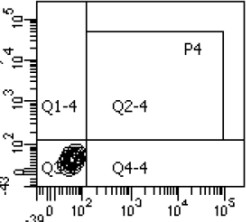


RORγt


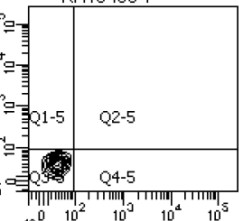


IL-17


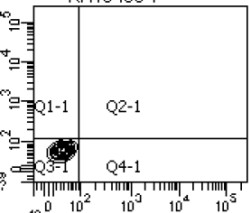


IL-17

SSC-A

FSC-H

DCD

T-bet

RORγt

**Supplementary figure 2. Gating strategy for modulation in cytokine, transcription factor, T helper subsets expression upon hormone stimulation in RA CD4+ T cells.** Representative flow cytometry plots shows isolated CD4+ T cells where lymphocyte population is gated first, followed by singlet population. Live cells are gated using Zombie violet dye. The dual positive population of cytokines and transcription factor specific for Th1 cells (T-bet+ IFNγ+) and Th17 cells (RORγt+ IL-17+) are analyzed for any modulation.

T-bet

IFNγ


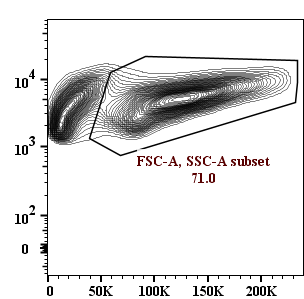


FSC-A


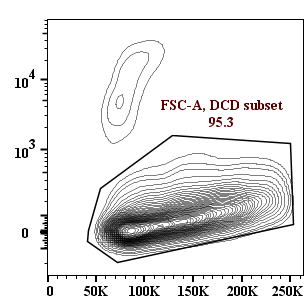


FSC-A


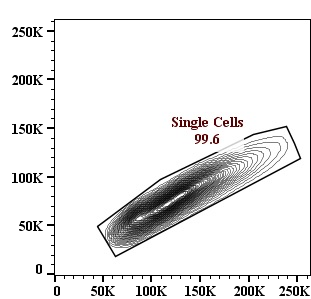


FSC-A


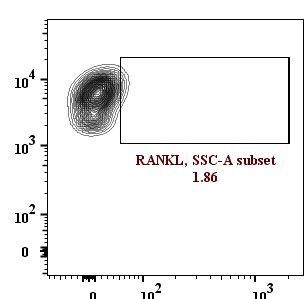


RANKL

V

V

SSC-A

FSC-H

DCD

SSC-A

V

V

FasL

**Supplementary figure 3. Gating strategy for modulation in RANKL and FasL expression upon hormone stimulation in RA CD4+ T cells.** Representative flow cytometry plots shows isolated CD4+ T cells where lymphocyte population is gated first, followed by singlet population. Live cells are gated using Zombie violet dye. Modulation in RANKL and FasL was then analyzed.

SSC-A


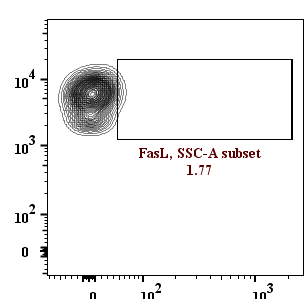


## Before Isolation


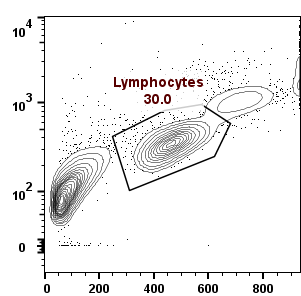


FSC-A


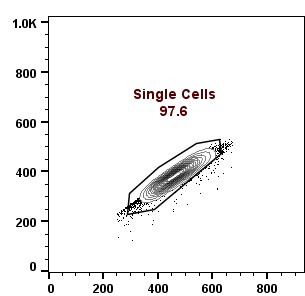


FSC-A


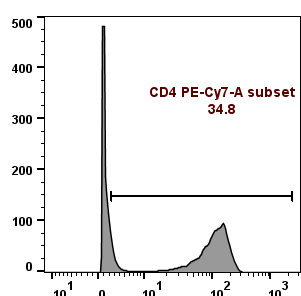


CD4+


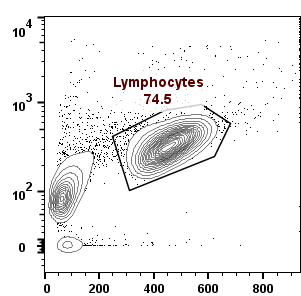


FSC-A


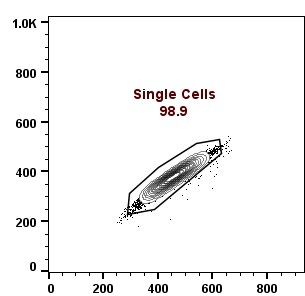


FSC-A


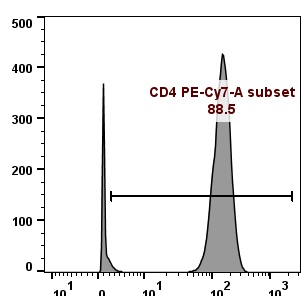


CD4+

SSC-A

FSC-H

Count

**After Isolation**

**Supplementary figure 4. CD4+ T cells purity validated prior *ex-vivo* experiments:** Representative flow cytometry plots shows purity of CD4+ T cells isolated from healthy control PBMCs prior ex-vivo Th1 and Th17 differentiation studies

SSC-A

FSC-H

Count


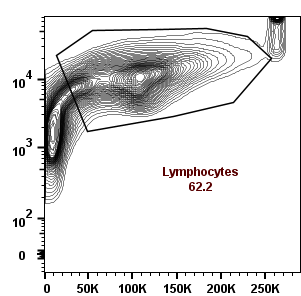


FSC-A


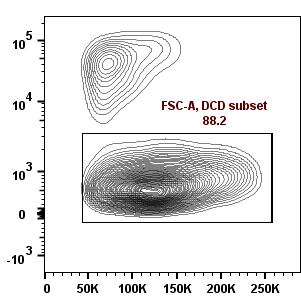


FSC-A


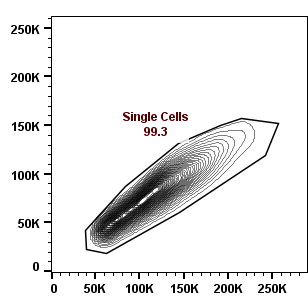


FSC-A

SSC-A

FSC-H

DCD

SSC-A

SSC-A

SSC-A

SSC-A

V

**Supplementary figure 5. Gating strategy for modulation in cytokine, transcription factor and T helper subset upon hormone stimulation in *ex-vivo* Th1 and Th17 cells.** Representative flow cytometry plots shows isolated CD4+ T cells where lymphocyte population is gated first, followed by singlet population. Live cells are gated using Zombie violet dye. Modulation in RANKL and FasL was then analyzed.


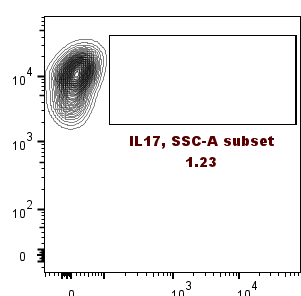

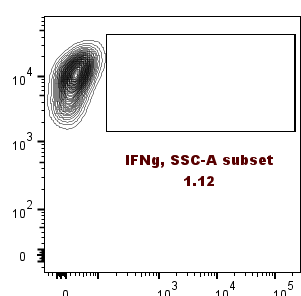

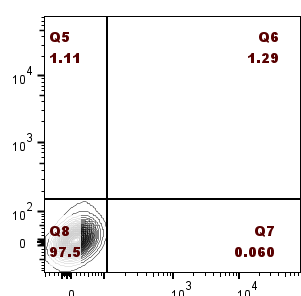


IFNγ

IL-17

IL-17


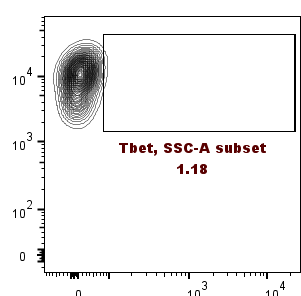

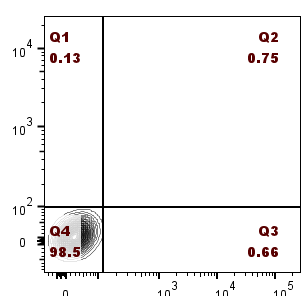

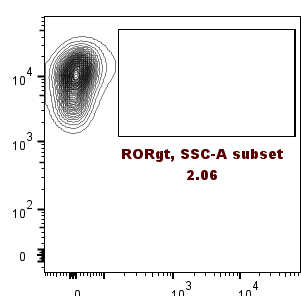


T-bet

RORγτ

IFNγ

T-bet

RORγt

**Supplementary Table 1. Reagents and software used in the study**

| **S.no** | **Reagent** | **Catalogue no.** |
| --- | --- | --- |
| 1. | Human ProcartaPlex Mix & Match 46-Plex kit | Invitrogen  PPX-46-MX324DE |
| 2. | ProcartaPlex Human Antibody Isotyping Panels | Invitrogen  EPX070- 10818-901 |
| 3. | ProTM Human Cytokine Screening 48-Plex Panel | Bio-Rad 12007283 |
| 4. | Histopaque-1077 | Sigma  10771 |
| 5. | Hyaluronidase | Sigma Aldrich H3506-100MG |
| 6. | Zombie fixable violet dye kit | BioLegend  423113 |
| 7. | RPMI 1640 | PAN-BIOTECH P04-16520 |
| 8. | Fetal Bovine Serum | PAN-BIOTECH  P30-1402 |
| 9. | DPBS, w/o: Ca and Mg | PAN BIOTECH P04-36500 |
| 10. | Dynabeads ^TM^ Untouched Human CD4 T cell kit | Invitrogen  11346D |
| 11. | Phorbol 12-myristate 13-acetate (PMA) | Sigma P8139-1MG |
| 12. | Ionomycin | Sigma  I0634-1MG |
| 13. | Brefeldin A (BFA) | Sigma B6542-5MG |
| 14. | β-Estradiol powder | Sigma  E2758-1G |
| 15. | Progesterone powder | Sigma P6149-1MG |
| 16. | BD Cytofix / Cytoperm ^TM^ | BD Biosciences 554714 |
| 17. | eBiosciences^TM^ FOXP3/ Transcription factor Staining Buffer Set | Invitrogen 00-5523-00 |
| 18. | Triton X | Sigma  T8787-250mL |
| 19. | Formaldehyde | Sigma  F8775-500mL |
| 20. | Methanol | Sigma  646377-1L |
| 21. | FlowJO Version 10.8 | BD Biosciences |
| 22. | GraphPad Prism 9 | Dotmatics Pvt ltd. |

| **S.no** | **Cytokines / Neutralizing antibodies** | **Catalogue No.** |
| --- | --- | --- |
| 1. | IL-12 Protein Human Recombinant | Thermo  200-12H-10UG |
| 2. | IL-2 Protein Human Recombinant | Prospec Bio CYT-209 |
| 3. | IL-1β Protein Human Recombinant | Prospec Bio CYT-208 |
| 4. | TGF-β Protein Human Recombinant | Prospec Bio CYT-716 |
| 5. | IL-6 Protein Human Recombinant | Prospec Bio CYT-213 |
| 6. | IL-21 Protein Human Recombinant | Prospec Bio CYT-408 |
| 7. | IL-23 Protein Human Recombinant | Thermo  200-23-10UG |
| 8. | InVivoMAb anti-human CD3 | BioXcell BE0001-2 |
| 9. | InVivoMAb anti-human/monkey CD28 | BioXcell BE0291 |
| 10. | InVivoMAb anti-human IL-4 | BioXcell BE0240 |
| 11. | InVivoMAb anti-human IFNγ | BioXcell  BEO235 |

| **S.no.** | **Fluorochrome- tagged antibody** | **Catalogue No.** | |
| --- | --- | --- | --- |
|  |  | **Antibody** | **Isotype** |
| 1. | CD3-AF700 | Biolegend 317340 | Biolegend  400248 |
| 2. | CD4-PerCPCy 5.5 | BioLegend  300530 | BioLegend  552834 |
| 3. | CD4- PECy7 | BioLegend 300512 | Invitrogen  25-4714-42 |
| 4. | CXCR3- AF488 | BioLegend 353710 | Invitrogen  53-4714-42 |
| 5. | CCR6- PECy7 | BD  560620 | Invitrogen  25-4714-42 |
| 6. | ERα-PE | CST 74244S | CST 5742S |
| 7. | PR A/B- AF647 | CST 55652S | CST 2985S |
| 8. | IFNγ- AF 647 | BioLegend 502516 | BD 557783 |
| 9. | IL-17- PerCPCy5.5 | BD  560799 | BioLegend  552834 |
| 10. | TNF-α- AF700 | BD  557996 | BD 557882 |
| 11. | RORγt- PE | BD  563081 | BD 559529 |
| 12. | T-bet- PECy7 | Invitrogen 25-5825-82 | Invitrogen  25-4714-80 |
| 13. | RANKL- APC | BioLegend 347507 | BioLegend  982108 |
| 14. | FasL- BV711 | BD  744101 | BD 563044 |
| 15. | pSTAT1- AF647 | BioLegend 686412 | BD  557732 |
| 16. | pSTAT3-PE | BioLegend  698906 | BioLegend  400322 |
| 17. | pSTAT5- PECy7 | Invitrogen  25-9010-42 | Invitrogen  25-4714-80 |
